# Engineering hope: tissue-engineered intestine as a breakthrough for pediatric short bowel syndrome

**DOI:** 10.1097/MS9.0000000000003788

**Published:** 2025-08-28

**Authors:** Ayesha Khan, Maliha Khalid, Muhammad Talha, Aminath Waafira

**Affiliations:** a Dow Medical College, Karachi, Pakistan; b Jinnah Sindh Medical University, Karachi, Pakistan; c King Edward Medical University, Lahore, Pakistan; d Department of Medicine, The Maldives National University, Malé, Maldives

**Keywords:** pediatric surgery, regenerative medicine, short bowel syndrome, tissue engineering

## Abstract

Short bowel syndrome (SBS) in children, most often caused by necrotizing enterocolitis, volvulus, intestinal atresia, or gastroschisis, results in severe nutrient malabsorption and is associated with high morbidity. Tissue-engineered small intestine (TESI) offers a regenerative alternative to transplantation, circumventing organ scarcity and the need for lifelong immunosuppression. Derived from patient-specific stem cells, TESI can structurally mimic native intestine and integrate functionally, as demonstrated in preclinical rat models showing near-complete weight restoration, prolonged transit time, preserved vitamin B12 levels, and absorptive maturation. TESI generated from human or mouse cells expresses essential enzymes such as sucrase-isomaltase and SGLT-1, supporting glucose absorption, though activity levels remain lower than native tissue. Major challenges include limited tissue yield, potential immune barriers, length constraints, and complex surgical integration. Despite these hurdles, TESI holds promise as a durable, functional cure for pediatric SBS, with successful translation dependent on interdisciplinary collaboration and robust clinical evaluation.

*To the Editor*,

Short bowel syndrome (SBS) is a condition marked by poor nutrient absorption due to the loss of a substantial segment of the small intestine, from either birth defects or surgical removal. In infants and children, frequent causes include necrotizing enterocolitis, midgut twisting (volvulus), congenital intestinal blockages (atresia), and abdominal wall defects like gastroschisis^[1]^. SBS is classified as a rare disorder, with an estimated incidence of 24.5 per 100 000 live births annually^[2]^.

Tissue-engineered small intestine (TESI) provides an intriguing alternative to conventional intestinal transplantation by avoiding the major drawbacks of donor organ scarcity and the requirement for lifelong immunosuppression. Since TESI is derived from the patient’s own stem cells and scaffold constructs, it bypasses both graft rejection and systemic immune suppression without relying on donor availability^[3]^.

In a rat model of massive bowel resection, TESI showed complete formation in all implanted animals and mimicked native intestinal architecture, including muscular and neuronal layers. TESI animals regained 98.5% of their preoperative weight by day 40 versus 76.8% in controls (*P* < 0.03), and had significantly longer transit times (1825 vs. 982 minutes; *P* = 0.0488). Serum vitamin B12 levels remained normal in TESI recipients but were deficient in controls (*P* = 0.0159). Gene expression of villin and IAP confirmed absorptive maturation. These findings demonstrate TESI’s structural and functional integration, supporting its promise in regenerative strategies for intestinal failure. Furthermore, the study validates TESI’s potential to maintain systemic nutritional markers and modulate gastrointestinal physiology effectively. It lays foundational evidence for future translation to clinical models^[4]^.

TESI, generated from human or mouse cells, shows promising functional properties as a potential treatment for short bowel syndrome. It expresses vital digestive enzymes such as sucrase-isomaltase and sodium-glucose co-transporter SGLT-1. Functional testing confirmed active sucrase and maltase enzymes, enabling breakdown of complex sugars into glucose. Although these activities were lower than in native intestine, the presence of essential digestive and absorptive machinery supports TESI’s clinical relevance^[3]^.

Although TESI shows promise, major translational hurdles remain. These include low tissue yield from donor intestine, risk of immune rejection, limited length gain, and complex surgical requirements. These challenges raise concerns about its real-world scalability and whether it offers significant benefits over conventional intestinal transplantation^[5]^.

As TESI approaches clinical application, progress hinges on strong interdisciplinary collaboration and rigorous clinical trials. For infants living with the lifelong challenges of short bowel syndrome, TESI represents more than scientific advancement, it offers real hope for a functional, durable cure that could transform their quality of life. Our work is in line with the TITAN Guidelines on the need for transparency in AI use in healthcare^[6]^.

## Data Availability

None.
